# Design and Validation of a High-Speed Miniaturized Thermocycler with Peltier Elements for Efficient PCR Thermal Cycling

**DOI:** 10.3390/s25227046

**Published:** 2025-11-18

**Authors:** Passar Bamerni, Jan König, Lee-Ann Mistry, Katrin Schmitt, Jürgen Wöllenstein

**Affiliations:** 1 Laboratory for Gas Sensors, Department of Microsystems Engineering-IMTEK, University of Freiburg, Georges-Köhler-Allee 102, 79110 Freiburg, Germany; jan.koenig@ipm.fraunhofer.de (J.K.); katrin.schmitt@imtek.uni-freiburg.de (K.S.); juergen.woellenstein@imtek.uni-freiburg.de (J.W.); 2 Fraunhofer Institute for Physical Measurement Techniques IPM, Georges-Köhler-Allee 301, 79110 Freiburg, Germany

**Keywords:** polymerase chain reaction, precise and fast thermal cycling, thermoelectric, heating, cooling

## Abstract

We present a high-speed, miniaturized, Peltier-driven thermocycler for Polymerase Chain Reaction (PCR) with heating rates of 22.25 °C/s and cooling rates of 5.30 °C/s, using a standard aluminum block (a four-well section of a 96-well plate) and laterally arranged micro-thermoelectric coolers (TECs) to induce predominantly horizontal heat flow. Simulations without copper preheating predict a cooling rate of 5.70 °C/s. Finite-element thermoelectric modeling (COMSOL 6.2) closely matches measurements. The selection of materials is guided by the introduction of the merit number G_β_ that balances thermal diffusivity and volumetric heat capacity, enabling consistent comparison across candidate block materials. The performance of this system is evaluated against data reported in scientific literature, encompassing both recent academic developments and selected commercial systems that employ silver components to enhance thermal conductivity. Despite aluminum’s lower thermal conductivity, our device achieves superior thermal cycling rates, demonstrating that with innovative design, less expensive materials can compete with higher-performing ones. This work includes detailed numerical simulations, comparative analyses of block materials, design considerations, fabrication methods, and experimental validation. By integrating insights from current scientific research, this study contributes to the advancement of accessible and high-performance diagnostic technologies.

## 1. Introduction

The Polymerase Chain Reaction (PCR) is a foundational method for amplifying specific DNA sequences in research, molecular medicine, and diagnostics [1]. However, traditional PCR methods are often time-consuming and heavily dependent on equipment, limiting their use in scenarios requiring rapid results or outside advanced laboratory settings. At the heart of the PCR process is a precise and fast thermal cycling, where the ability to quickly switch between different temperatures is crucial for the efficiency and speed of DNA amplification. Conventional thermocyclers used for this purpose are typically large, slow, and unsuitable for portable applications, representing a significant barrier to the widespread adoption of PCR technology [1,2,3,4,5].

Although numerous current research projects show the great potential of miniaturized and portable thermal cyclers with innovative heating concepts, these approaches are often accompanied by considerable disadvantages: the complexity of the system design, the limited reproducibility and high manufacturing costs due to the usage of silver continue to pose key challenges. For example, inductive heating systems such as that of Xie et al. [6] require special magnetic coil designs and an integrated water-cooling system, while photothermal processes with plasmonic nanoparticles [7] require complex material preparation and precise optical control. Although thin-film heating elements on polymer chips [8] and PCB-based platforms [9,10] offer compact and sometimes cost-effective solutions, they are often limited in terms of temperature homogeneity, scalability and integration into existing laboratory infrastructures.

In contrast, conventional thermal cyclers typically rely on aluminum or copper blocks in combination with Peltier elements and PID control. These enable moderate heating and cooling rates of around 2.8 to 4 °C/s, such as those achieved by the high-speed mini-cycler from Just et al. [11], the portable RT-PCR system from Chong et al. [12], or the particularly cost-efficient solution from Sun et al. [13]. Manufacturers like SensoQuest, Analytik Jena, Qiagen, and Eppendorf improve these values by using silver blocks, which enable cooling rates of up to 5.5 °C/s and heating rates of up to 8 °C/s due to their significantly higher thermal conductivity compared to aluminum [14,15,16,17]. Table 1 lists the respective PCR devices with their heating rates, cooling rates, and costs.

A decisive advantage of modern developments also lies in the availability of high-performance, miniaturized Peltier elements, which deliver remarkable heating and cooling performance with very compact dimensions (<10 × 10 mm) and high energy efficiency at the same time. Their low thermal mass, direct coupling to the reaction chamber and the possibility of precise, segmented control makes them ideal for locally differentiated temperature management, such as when processing several samples in parallel. Furthermore, their use does not require any complex additional infrastructure, which considerably simplifies their integration into portable and modular thermal cyclers. However, it is important that such elements must be carefully qualified, particularly regarding long term stability, temperature homogeneity on a microscale and their thermal coupling to the sample chamber. To exploit these potentials in a targeted manner, this study presents a novel, miniaturized thermal cycler that fits seamlessly into the standardized layout of conventional 96-well PCR plates. For practical simplification and better feasibility, a section of four wells was extracted from a standard plate and used for the setup.

Since thermal performance is a key design criterion, the following section explores the governing thermodynamic principles and material parameters that influence the system architecture.

## 2. Thermal Properties and Material Selection

Heating and cooling rates, also known as ramp rates, are critical performance metrics for PCR cyclers as they directly affect the efficiency, speed, and quality of temperature cycling. The amount of heat *Q* required to change the temperature of a material, or the reaction chamber is determined by its specific heat *c* [J/g·K], mass *m* [g], and temperature change Δ*T*, as expressed in the following equation:

(1) Q=m⋅c⋅∆T.

The Fourier Equation (2) describes the heat flow rate *dQ/dt* within the material as a function of its cross-sectional area *A*, thermal conductivity κ and the temperature gradient *dT/dx* along the material:

(2) $$\frac{dQ}{dt}=\kappa \cdot A\cdot \frac{dT}{dx}.$$

Equation (2) shows that the thermal conductivity of the material, along with its geometry and defined temperature gradients, is decisive for the heat flow and thus the heat supply and dissipation within the medium. A high thermal conductivity is therefore decisive for the rapid, homogeneous distribution of heat within a material. In thermoelectric materials, the thermal conductivity (*κ*) results from the sum of contributions from both electrons (*κ_e_*) and phonons (*κ_p_*) [24].

(3) $$\kappa =\kappa_{e}+\kappa_{p}.$$

In metals, electrons are primarily responsible for the heat transport [25], whereas the transport by phonons dominate the transport in dielectrica.

The Wiedemann–Franz law (Equation (4)) describes the relation of the thermal conductivity by electrons with the specific electrical conductivity *σ* [S/m]. The Lorentz number *L* can be estimated for most metals by approximately 2.44 × 10^−8^ WΩK^−2^ [24,25]. *T* is the absolute temperature [K].

(4) $$\kappa_{e}=L\cdot \sigma \cdot T.$$

The choice of material is a fundamental factor in optimizing heating and cooling rates [26]. Table 2 highlights that silver, with a thermal conductivity of 429 W/m·K, has an 81% higher thermal conductivity than aluminum. This difference is mainly due to the higher electron mobility and the lower number of electrons per atom in the valence band of silver. Aluminum, with atomic number 13, has an electron configuration of [Ne]3s^2^3p^1^, providing three valence electrons in the 3s and 3p orbitals [27,28]. In contrast, silver has only one valence electron with an electron configuration of [Kr] 4d^10^5s^1^ [29].

The electron mobility of aluminum is 36 cm^2^/Vs, whereas silver shows a significantly higher mobility of approximately 62 cm^2^/Vs, partly because in aluminum the electrons are more tightly bound within the lattice. The electron density in silver is 5.86 × 10^28^ electrons/m^3^, much lower than in aluminum with 2.1 × 10^29^ electrons/m^3^. Although the electron density affects the electrical conductivity directly, the overall electrical conductivity σ results from the electron density n, the elementary charge e, and the electron mobility µ as described by Equation (5):

(5) σ =n⋅e⋅μ. 

The electrical conductivity of silver (σ_Ag_ = 58.2 × 10^6^ S/m) is significantly higher than that of aluminum (σ_Al_ = 12.1 × 10^6^ S/m), which also enhances its thermal conductivity [24]. The specific heat capacity of silver is 235 J/kg·K, which is 74% lower than that of aluminum. This lower heat capacity is attributed to the higher atomic mass of silver atoms (107.86 u) compared to aluminum atoms (26.9 u) [30,31]. As a result, fewer atoms per gram need to be heated, requiring less energy to raise the temperature. Additionally, the higher atomic mass of silver reduces defect formation in its face-centered cubic lattice, resulting in lower scattering of both electrons and phonons, which further enhances its thermal conductivity [32].

**Table 2:** Materials and their corresponding thermal conductivities and specific heat capacities at a temperature of ca. 25 °C.

	Thermal Conductivity *κ* [W/m·K]	Specific Heat Capacity c_p_ [J/kg·K]	Density *ρ* [kg/m^3^]
Al_2_O_3_	35 [33]	880 [30]	3940 [33]
AlN	170 [34]	740 [35]	3260 [36]
Al	237 [25]	900 [31]	2700 [37]
Au	315 [31]	128 [31]	19,320 [38]
Cr	94 [37]	448 [37]	7190 [37]
Cu	401 [25]	386 [25]	8940 [37]
Ag	429 [25]	235 [31]	10,490 [37]

To mitigate the oxidation of silver, blocks are often coated with gold, which serves as a protective barrier without significantly impacting thermal conductivity [39]. The lower specific heat capacity of silver leads to higher heating and cooling rates in thermal cyclers [31].

In order to establish a uniform foundation for the analysis of material trends, the concise merit number *G_β_* is proposed for the well material in question. In the case of identical geometry and power, the ramp is governed by two factors: wall diffusion and the energetic mass of the block. Materials differ only by the values of *κ*, *ρ*, and *c_p_*. The combination of an energetic index and a diffusion index results in the calculation of a scalar merit. Both indices are normalized to the standard material aluminum used in the PCR:

(6) $$G_{\beta }={\left(\frac{(\rho c_{p}{)}_{Al}}{(\rho c_{p}{)}_{M}}\right)}^{1-\beta }\cdot {\left(\frac{\frac{\kappa_{M}}{(\rho c_{p}{)}_{M}}}{\frac{\kappa_{Al}}{(\rho c_{p}{)}_{Al}}}\right)}^{\beta }=\frac{(\rho c_{p}{)}_{Al}}{(\rho c_{p}{)}_{M}}\cdot {\left(\frac{\kappa_{M}}{\kappa_{Al}}\right)}^{\beta },\;\;\;\;\;\;\;\;0\leq \beta \leq 1\;.$$

In this formula, *κ* denotes the thermal conductivity, *ρ* the density, and *c_p_* the specific heat capacity of the respective material *M*. The β-weighting factor is used to calculate the diffusion fraction relative to the energetic mass. Here, *β* = 0 corresponds to the power-limited classification (*ρc_p_*)^−1^ and *β* = 1 corresponds to the diffusion-limited classification *κ*(*ρc_p_*)^−1^. Accordingly, a small *β* is selected for operation with short heat paths and a high *β* for high contact resistances. The relationship between *β* and the physical structure can be described by the characteristic distance *L*. It quantifies the dominant heat transport path in the system. The distance *L* is the path that heat travels from the source, i.e., the hot or cold surface of the Peltier element, to the target zone, such as a PCR well. It can be understood as the average length of the temperature gradient *dT/dx*, i.e., the range over which a relevant temperature difference develops in the material. Empirically and physically, *β* is approximately proportional to the heat transport distance (*β* ∝ *L/L_ref_*). *L_ref_* is a reference distance at which the system still operates in a power-dominated manner.

For short heat paths of less than 2 mm, such as direct contact between the Peltier element and the aluminum block, the *β* values are between 0.1 and 0.2, which corresponds to power-dominated behavior. Average distances of 2–5 mm with moderate contact resistances result in *β* values of 0.3–0.5. For longer paths of 5–10 mm or higher contact resistances, the process becomes increasingly diffusion-limited (*β* = 0.6–0.8). In strongly diffusion-dominated cases with lateral transport paths exceeding 10 mm, *β* can rise to around 1.0. This classification is based on simulations and empirical experience with heat paths in Peltier-based mini-thermocyclers. It describes the characteristic length over which temperature homogeneity in the material is achieved mainly through heat conduction and no longer through direct power supply. Based on these theoretical foundations, a concrete system concept was developed. The following section describes the technical construction of the thermocycler, with particular focus on the arrangement of the Peltier elements and their thermal integration.

## 3. Design and Performance

### 3.1. Materials and Equipment

The initial model of the miniaturized thermocycler was assembled from commercially available components and custom-machined metal blocks. The primary materials and instruments utilized in this study are listed below to ensure replicability.

#### 3.1.1. Materials

Commercial Bi_2_Te_3_-based thermoelectric (Peltier) coolers (Ferrotec GmbH, Unterensingen, Germany) were used as heating and cooling units. Thermal coupling between the copper plates and the reaction block was achieved using a self-adhesive graphite mounting foil QGF-G02AA (Quick-Ohm GmbH, Herford, Germany) with high in-plane thermal conductivity. The reaction chamber consisted of an aluminum segment taken from a standard 96-well PCR plate (Eppendorf AG, Hamburg, Germany). Oxygen-free copper plates (OFHC) served as thermal reservoirs. Mechanical assembly employed non-magnetic stainless-steel screws and spacers (DIN 912, A2-70), with polyimide insulation film (Kapton^®^ HN, DuPont de Nemours Inc., Wilmington, NC, United States) and high-temperature epoxy adhesive (Loctite 9492, Henkel AG & Co. KGaA, Düsseldorf, Germany) to ensure electrical isolation and mechanical stability.

#### 3.1.2. Equipment

Electrical power for the Peltier modules was provided by a programmable DC supply HMP4040 (Rohde & Schwarz GmbH & Co. KG, Munich, Germany). Temperature monitoring employed type-K thermocouples (Omega Engineering Inc., Norwalk, CT, USA) connected to a data logger 34972A (Keysight Technologies Inc., Santa Rosa, CA, USA). AC resistance and Seebeck voltage measurements were carried out using a function generator AFG1022 and a precision multimeter 34461A (Keysight Technologies Inc.). Data acquisition and device control were implemented through a USB-DAQ NI USB-6003 (National Instruments Corp., Austin, TX, USA) operated via custom Python 3.13.0 scripts. Finite-element simulations were performed in COMSOL Multiphysics^®^ 6.2 (COMSOL AB, Stockholm, Sweden) using the Heat Transfer and Thermoelectric modules. All experiments were conducted at room temperature (22 ± 1 °C) under still-air conditions.

#### 3.1.3. Cost Basis

The material cost reported for “This work” (161 USD) represents a single-unit bill of materials and excludes shared laboratory infrastructure such as the power supply, data acquisition unit, and data logger. The commercial system prices listed in Table 1 correspond to indicative distributor or list prices for silver-block configurations employed in the referenced industrial products [14,15,16,17]. All values are expressed in USD, converted using the reference exchange rates provided in the table notes.

### 3.2. Prototype Design and Assembly

**Figure 1:**
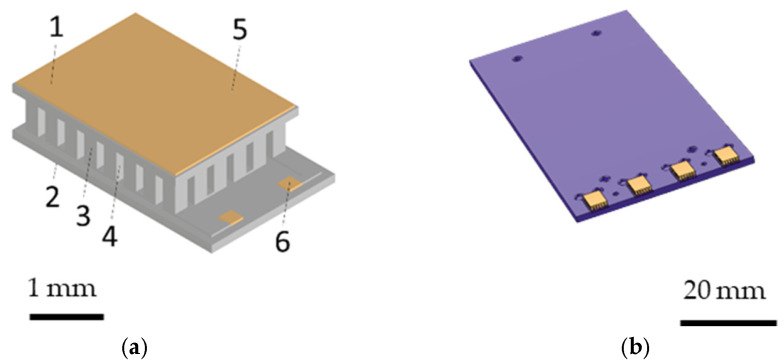
(**a**) Peltier elements made of Bi_2_Te_3_ used for heating and cooling the miniaturized prototype thermocycler. (**b**) Copper plate equipped with Peltier elements and simulated in COMSOL 6.2.

The prototype of the miniaturized thermal cycler developed in this study consists of eight Peltier elements, a graphite foil cut to size for attachment to two copper plates and a commercially available aluminum block cut to the size of four reaction chambers. Figure 1a shows the Peltier element (Ferrotec) used in this design, with a base area of 5.5 mm × 3.4 mm and a height of 1.13 mm. Each Peltier element consists of 24 n-doped and 24 p-doped bismuth telluride (Bi_2_Te_3_) legs, which are fixed by two parallel aluminum oxide plates. Both contact surfaces of each Peltier element are coated with a 500 nm thick gold layer. The upper substrate is heated or cooled depending on the current direction, while the lower substrate performs complementary cooling or heating. The materials and structural components used in the Peltier element are summarized in Table 3, including the semiconductor legs, substrates, and metallization layers. The copper plates depicted in Figure 1b each weigh 10 g, have an area of 70 × 36 mm, a depth of 1.5 mm and serve as a thermal mass. 

The heat capacity per copper plate is therefore 3.85 J/K. The copper plates each have four recesses for Peltier elements, which are attached to the respective plate recess with a graphite heat-conducting adhesive. The self-adhesive graphite thermally conductive mounting foil is 0.125 mm thick and has an adhesive layer of 22 µm on both sides. The thermal conductivity of the film is 5–16 W/m·K across the fiber or through the surface and 155–470 W/m·K in the direction of the fiber, i.e., in the surface [40].

This design differs from previous PCR systems primarily in that the Peltier elements are positioned parallel to the well depth of the aluminum block, enabling heat transport primarily in a horizontal plane rather than vertically, as observed in conventional designs listed in Table 1. The aluminum chamber has a mass of 4.4 g and a heat capacity of 3.96 J/K. Due to the small dimensions of the Peltier elements, it is possible to use these Peltier elements for horizontal temperature control of commercially available aluminum tanks. For this purpose, the four Peltier elements per copper plate are pressed against the planar aluminum blocks. The holes in the parallel copper plates are used to fix them in place using screws. The four Peltier elements per copper plate are thermally connected in parallel and electrically connected in series.

**Figure 2:**
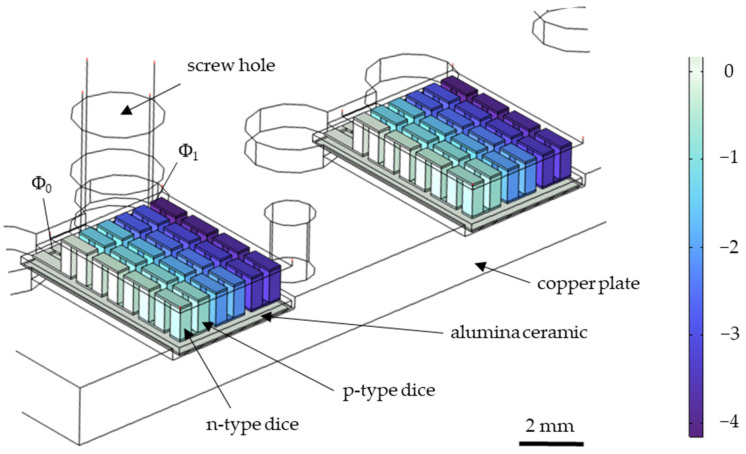
Simulation of the electrical potential within the Peltier elements with a Voltage Scale Indicator. The upper alumina substrate of the Peltier elements has been removed in the simulation view for better visualization.

Figure 2 shows the individual Bi_2_Te_3_ legs of two Peltier elements, each applied to a copper plate. By removing the upper alumina substrate in the COMSOL 6.2 simulation, it can be clearly seen how the electrical potential is evenly distributed in blue over the Bi_2_Te_3_ legs connected in series when a voltage of U_P_ = Φ_1_ − Φ_0_ = 4 V is applied to each Peltier element. The observed uniform voltage drop across the Peltier element can be attributed to the identical resistance presented by each thermocouple connected in series. This is essential to prevent any temperature gradient or hotspot within a Peltier element during the heating and cooling process, as demonstrated in Equation (7) where Q denotes the thermal energy generated, U the applied voltage, R the electrical resistance, and t the time duration over which the voltage is applied.

(7) $$Q=\frac{U^{2}}{R}\cdot t.$$

Hotspots can frequently occur if these parameters are not optimally managed or the element experiences uneven loading [41].

### 3.3. Electrical Characterization and Preliminary Tests

Before the individual components are brought together, practical preliminary tests must be carried out on the Peltier elements to ensure proper operation. The Peltier elements must have approximately the same resistance values due to the electrical series connection. For this purpose, an alternating current I_AC_ with a frequency of 10 kHz is applied. As a result, the Peltier effect becomes negligibly small, so that only the ohmic resistance R_Ohm_ remains.

(8) $$R_{Ohm}=\frac{V_{AC}}{I_{AC}}.$$

**Figure 3:**
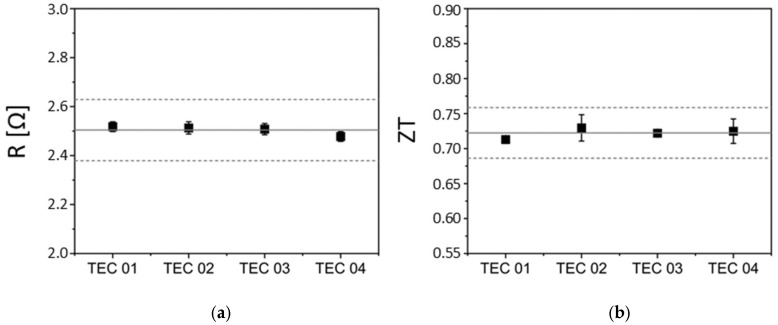
(**a**) Experimentally determined ohmic resistance values; (**b**) Experimentally determined thermoelectric figure of merit (*ZT*) of the Peltier elements, also known as Thermo-Electric Coolers (TEC).

The resulting voltage V_AC_ is measured and used to calculate the ohmic resistance R_Ohm_ according to Equation (8). Deviation limits of ±5% are set for assessing the suitability of the Peltier elements as electronic components [42]. Figure 3a shows that four tested Peltier elements of a copper plate are within the deviation of ± 5%, confirming their suitability for electrical use.

The thermoelectric figure of merit *ZT* is dimensionless and allows for the comparison of the performance levels of different thermoelectric materials.

(9) $$ZT=\frac{\alpha^{2}\cdot \sigma \;}{\kappa }T=\frac{V_{Seebeck}}{V_{Ohm}}\;.$$

This is determined using the Seebeck coefficient *α*, the electrical conductivity *σ*, and the thermal conductivity *κ* [43]. *Harman* demonstrated that the thermoelectric figure of merit *ZT* can be directly determined from the ratio of the Seebeck (*V_Seebeck_*) to the Ohmic (*V_Ohm_*) voltage, without the need for separate measurements of thermal conductivity, electrical conductivity, and Seebeck coefficient [44]. Figure 3b shows the individual *ZT* values of four Peltier elements. The maximum deviation in relation to the mean value of all Peltier elements is 1.3%, which is within the ±5% limits entered. The Bi_2_Te_3_-based Peltier elements used in this work achieve a *ZT* value of approximately 0.72 at room temperature, which is in line with the typical performance of bulk bismuth telluride thermoelectrics, known to reach around 0.70 at 25 °C [45]. A four-point measurement technique was employed to eliminate voltage drops at contact resistances and to minimize the influence of Joule heating on the electrical measurements. The *ZT* value is calculated purely electrically using the Seebeck voltage, current, and electrical resistance, making direct temperature measurement unnecessary. However, the underlying temperature difference across the Peltier element must still be implicitly assumed or estimated based on known material parameters. This introduces several potential sources of uncertainty. These include assumptions regarding thermal boundary conditions, possible inhomogeneous temperature distributions, and reliance on nominal material parameters such as the Seebeck coefficient α and thermal conductivity. Additionally, heat losses to the environment (e.g., through radiation or convection) and incomplete thermal coupling can affect the actual temperature distribution within the element and thereby systematically distort the measured Seebeck voltage.

**Figure 4:**
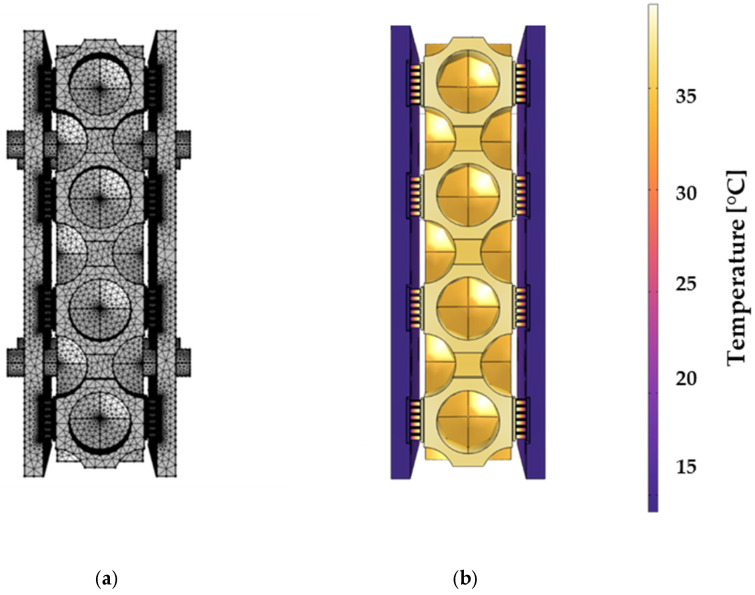
Simulation of the prototype thermal cycler investigated in this work: (**a**) Mesh-up; (**b**) Temperature distribution in °C.

The successful preliminary tests of the Peltier elements facilitate the assembly and operation of the components as shown in Figure 4.

### 3.4. Numerical Simulation

The complete assembly is subjected to thermoelectric analysis using the finite element method (FEM) in COMSOL 6.2 software. For this purpose, the geometric space is discretized into a mesh of tetrahedral elements, as illustrated in Figure 4a. The densities of the meshes vary along the structure for various reasons. A finely resolved mesh increases the accuracy of the simulation through more precise modeling of complex physical phenomena and their numerical solution [46]. Complex geometries, such as corners and fine details, require finer meshes for accurate representation. In contrast, simpler shapes can utilize coarser meshes. For physical phenomena with significant gradients, like high temperature differentials or rapid velocities, increased mesh density is essential for precision. Conversely, areas with minimal physical gradients can employ coarser meshes without compromising accuracy. COMSOL’s adaptive meshing techniques automatically adjust densities based on expected error levels, optimizing model precision. The model is simulated in heating mode with room temperature as the starting condition, with natural convection and a heat transfer coefficient of 5 W/m^2^·K [47]. In addition to the material-dependent conduction, heat is transferred by radiation, which is defined by the geometry and the material-specific emissivity ε [48].

**Figure 5:**
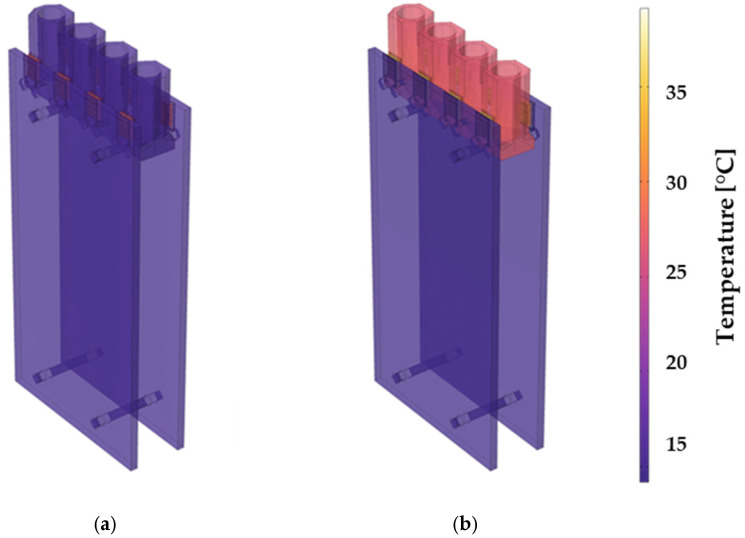
Simulation of the temperature distribution of the thermal cycler during the heating process (**a**) at the start, (**b**) after a few seconds.

Figure 4b shows the simulation of the temperature distribution after heating the prototype for 2 s. While the hot substrate sides of the Peltier elements heat the aluminum chamber, the copper plates are cooled. Figure 5 demonstrates that both the copper plates and the aluminum block achieve homogeneous cooling and heating due to their high thermal conductivities (see Table 2). This is particularly important for thermal cyclers, as temperature imbalances in the well would impair the polymerase chain reaction. During the primer hybridization step, too high or too low temperature prevents efficient binding of the primers to the target DNA [49]. As expected, the largest temperature gradients are located within the bismuth telluride legs of the Peltier elements, as the low thermal conductivity thermally isolates the hot and cold sides from each other and ensures a high thermoelectric figure of merit according to Equation (9).

Following the construction of the prototype, the system’s performance is now validated. For our miniaturized design, which is power-dominated, we set β = 0.1 for all material comparisons. Both simulation data and experimental results are presented and critically discussed, with the aim of assessing the practical applicability of the design and comparing it to established systems.

## 4. Results and Discussion

**Figure 6:**
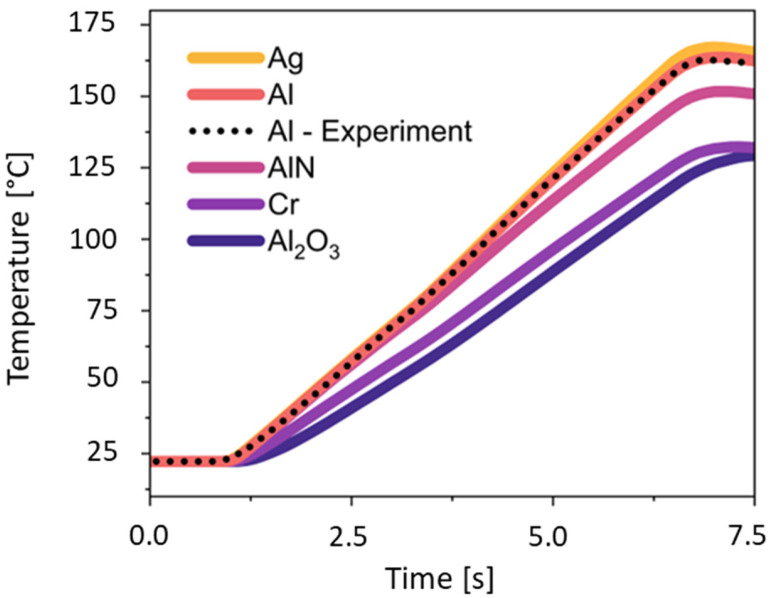
Simulation and experimental results within the block chamber of the thermal cycler for different materials during the heating process.

Figure 6 shows the simulated and experimental heating curves of the aluminum block. The heating process begins after one second at room temperature (~22 °C) and rises rapidly, demonstrating the immediate effect of the Peltier elements on the block. After 7 s, active heating ceases temperatures of around 163 °C, and both simulated and experimental temperature measurements stabilize. The diagram shows that the measured temperature values of the aluminum block confirm the results simulated with COMSOL 6.2.

Further investigations were conducted using other materials to assess their compatibility with the existing structure. Ceramics like Al_2_O_3_ and AlN, as well as metals such as Cr and polished Ag, were tested as alternatives to the aluminum block. The material-specific ranking of the merit factor G_M_ for *β* = 0.1 is confirmed by measurement and simulation results: G_Ag_ = 1.046 > G_Al_ = 1.000 > G_AlN_ = 0.974 > G_Cr_ = 0.688 > G_Al2O3_ = 0.579. Al_2_O_3_ exhibited a slower heating rate and a lower maximum temperature. The low thermal diffusivity of Al_2_O_3_ is primarily due to its low thermal conductivity of 35 W·(m·K)^−1^. Its density of about 3940 kg·m^−3^ and its specific heat capacity of 880 J·(kg·K)^−1^ increase volumetric heat storage (cf. Table 2). As a result, temperature gradients decay slowly.

Silver exhibits a thermal behavior similar to aluminum. The reason is the nearly identical volumetric heat capacities *ρc_p_*, i.e., the product of density *ρ* and specific heat capacity *c_p_*. The volumetric heat capacity of silver is approximately 10.490 kg·m^−3^ × 235 J·kg^−1^·K^−1^ ≈ 2.47 MJ·m^−3^·K^−1^. The volumetric heat capacity of aluminum is approximately 2.700 kg·m^−3^ × 900 J·kg^−1^·K^−1^ = 2.43 MJ·m^−3^·K^−1^. The curves diverge for T > 100 °C, with silver heating faster than aluminum. This occurs because polished silver has a markedly lower emissivity ε than aluminum. The radiative heat loss scales as

(10) $$q_{rad}=\varepsilon \cdot \sigma \cdot {(T}^{4}-T_{amb}^{4})\;.$$

Here *q_rad_* is the radiative heat flux [W·m^−2^], *ε* the emissivity, *σ* the Stefan–Boltzmann constant (5.67 × 10^−8^ W·m^−2^·K^−4^), *T* the surface temperature [K], and *T_amb_* the ambient temperature [K].

Consequently, aluminum absorbs more energy per degree of temperature change at higher temperatures compared to silver. Since the temperature range of a PCR is below 100.0 °C, these disadvantages of aluminum are negligible, provided that contact resistance between the sample and the aluminum chamber remains minimal. With aluminum as the block material, the prototype still achieves a heating rate of 22.25 °C/s. To ensure comparability, we explicitly define the temperature window for determining the heating rate as 30 °C to 100 °C. Apart from the high electrical insulation, which minimizes the risk of short circuits, AlN reaches high temperatures within a few seconds. The fact that volumetric heating capacity alone is not decisive for performance is clearly illustrated by the example of AlN, which, at 2.41 MJ·m^−3^·K^−1^, is lower than that of aluminum. The merit index G_β_ developed in this work takes the necessary thermal diffusivity into account. Alongside its high thermal conductivity, AlN’s low coefficient of thermal expansion [50], chemical resistance, and biocompatibility [51] provide important conditions for its use as a ceramic block material for PCR.

**Figure 7:**
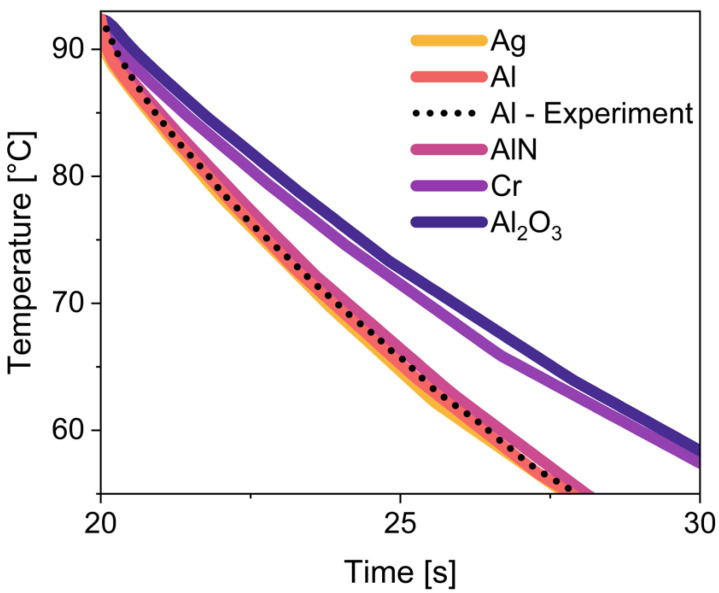
Cooling rates in the block chamber of the miniaturized thermal cycler, simulated for different materials and experimentally determined for aluminum.

The cooling rates of the miniaturized thermal cycler have been simulated for several materials in Figure 7 and verified experimentally for aluminum. As a starting condition, the copper plate was preheated to a temperature of 47 °C in order to simulate the performance of the cooling process after many cycles and thus more difficult conditions. Nevertheless, the thermal cycler designed in this work with Al as the reaction chamber achieved a cooling rate of 5.3 °C/s. The temperature window for determining the cooling rate is defined as 90 °C to 60 °C. If the copper preheating is neglected and the room temperature of 22.25 °C is assumed, cooling rates of 5.7 °C/s are even achieved in the simulation. Based on Equations (1) and (7), this result indicates that an increase in the thermal copper mass would increase the performance of the overall structure. The reason for this is the lower temperature rise with an increase in heat capacity, so that effective heat transport is guaranteed. The cooling curves of Al_2_O_3_ show the lowest cooling rate of 3.4 °C/s, followed by Cr with 3.5 °C/s, AlN with 4.5 °C/s, and Al and Ag with 5.3 °C/s, the highest cooling rate, which can be attributed to their thermal parameters from Table 2. The reason why Ag, despite its lower specific heat capacity and higher thermal conductivity, performs almost identically to Al in terms of cooling behavior is the high density of silver, which, at 10,490 kg/m^3^ [35], is almost four times that of aluminum at 2700 kg/m^3^ [35]. In the simulation, the volume of the individual components is kept constant, so that the advantages of silver are compensated for by its high density and, according to Figure 7, it corresponds approximately to the thermal properties of aluminum. The test results demonstrate that intelligent design can serve as a viable alternative to expensive high-performance materials.

Although the thermocycler presented here demonstrates excellent heating and cooling performance, some limitations of the current design should be taken into account. Long-term operation can impair the stability and performance of the Peltier elements due to, for example, solder joint fatigue, degradation of the contact surfaces, or thermomechanical stresses, which can lead to increased contact resistance or reduced efficiency in the long term. In addition, the thermal interface materials used, including graphite foil, can exhibit aging effects such as drying out or deformation, which can alter the thermal coupling during operation. Although the current four-well design offers advantages for prototype development and precise thermal characterization, it limits sample throughput. Scaling the current four-well design to higher formats, such as 24- or 96-well layouts, requires adapted power and temperature distribution, more mechanically uniform fixation, and improved heat dissipation to maintain homogeneity and ramp performance at the same level. These aspects will be further investigated as part of future design optimizations. The next step will be to carry out PCR experiments to validate the thermocycler’s practical performance and temperature uniformity under real amplification conditions.

## 5. Conclusions

The success of our thermal cycler lies in a holistic design approach that goes beyond the mere selection of high-conductivity materials. By strategically reducing thermal mass and optimizing the heat transfer interface through the integration of compact, high-performance Peltier elements, we achieved average heating rates of 22.25 °C/s and cooling rates of 5.30 °C/s using a commercially available, custom-sized aluminum block. These results were realized without the need for expensive or specialized materials like silver, underscoring the effectiveness of intelligent thermal design over costly raw material substitution. Complementary simulation studies comparing aluminum and silver blocks confirmed the strong impact of thermal conductivity on dynamic performance but also demonstrated that this limitation can be overcome by structural optimization and efficient coupling. The new merit index *G_β_* combines diffusion and energetic mass into a device-agnostic measure, enables reproducible material comparisons and the targeted design of thermocyclers, and is confirmed by simulations and measurements. Furthermore, additional simulations suggest that increasing the thermal mass of adjacent copper plates can further improve cooling performance without compromising speed or portability. Overall, our findings show that it is possible to rival and even exceed the thermal performance of state-of-the-art systems through a smart combination of material efficiency, component integration, and architectural innovation. This approach not only challenges existing industry standards but also provides a robust and cost-effective alternative for rapid and scalable PCR, particularly well-suited for laboratories with limited budgets and applications requiring a high throughput.

## Figures and Tables

**Table 1:** Published Heating and Cooling Rates of Thermal Cyclers.

PCR Device	Heating Rate [°C/s]	Cooling Rate [°C/s]	Cost [USD]	Source
Xie et al. (2024)	14.9	13.4	--	[6]
Cheong et al. (2020)	13.1	4.9	--	[7]
Jeong et al. (2018)	1.0	0.9	--	[8]
Kaprou et al. (2020)	1.4	0.6	--	[9]
Oliveira et al. (2021)	2.0	2.0	--	[10]
Just et al. (2023)	2.8	--	--	[11]
Chong et al. (2017)	3.0	--	2665	[12]
Sun et al. (2023)	4.0	8.1	170	[13]
Labcycler 48s	5.0	5.0	4790	[14]
Biometra TAdvanced 96 S	8.0	5.5	11,200	[15]
QIAquant 96	8.0	5.5	10,500	[16]
Mastercycler^®^ X50	10.0	5.0	9800	[17]
Open PCR	1.0	1.0	499	[18]
miniPCR^TM^ mini 16	2.4	1.7	749	[19]
Luo et al. (2025)	2.8	2.2	120	[20]
Yeom et al. (2022)	21.9	1.4	--	[21]
Ling et al. (2023)	6.1	5.3	--	[22]
Mandal et al. (2024)	2.0	--	120	[23]
This work	22.25	5.3	161	--

**Table 3:** Materials and components of the Peltier element used in this study.

Number	Component	Material	Quantity
1	hot side substrate	Al_2_O_3_	1
2	cold side substrate	Al_2_O_3_	1
3	n-type dice	Bi_2_Te_3_	24
4	p-type dice	Bi_2_Te_3_	24
5	metallization	Cu & Ni	2
6	metallization	Au	2

## Data Availability

Data can be obtained from the corresponding author upon request.
